# Novel Pyroptosis-Related Gene Signatures Identified as the Prognostic Biomarkers for Bladder Carcinoma

**DOI:** 10.3389/fonc.2022.881860

**Published:** 2022-06-30

**Authors:** Jia You, Huawei Li, Yuanfeng Wei, Peng Fan, Yaqin Zhao, Cheng Yi, Qing Guo, Xi Yang

**Affiliations:** ^1^ Department of Oncology, Hospital of Chengdu University of Traditional Chinese Medicine, Chengdu University of Traditional Chinese Medicine, Chengdu, China; ^2^ Department of Medical Oncology, Cancer Center, West China Hospital, Sichuan University, Chengdu, China; ^3^ Department of Respiratory and Critical Care Medicine, Chongqing Traditional Chinese Medicine Hospital, Chongqing, China; ^4^ Department of Oncology, Taizhou People’s Hospital, Taizhou, China

**Keywords:** bladder carcinoma, pyroptosis, prognostic biomarkers, immune infiltration, Lasso-Cox regression

## Abstract

**Background:**

Bladder carcinoma (BLCA) is a common malignant tumor with high morbidity and mortality in the urinary system. Pyroptosis is a pattern of programmed cell death that is closely associated with progression of tumors. Therefore, it is significant to probe the expression of pyroptosis-related genes (PRGs) in BLCA.

**Methods:**

The differentially expressed genes in normal and BLCA tissues were first obtained from the Cancer Genome Atlas (TCGA) database analysis, as well as PRGs from the National Center for Biotechnology Information (NCBI) database, intersecting to obtain differentially expressed pyroptosis-related genes (DEPRGs) in BLCA. With the construction of a prognostic model of pyroptosis by regression analysis, we derived and validated key genes, which were ascertained as a separate prognostic marker by individual prognostic and clinical relevance analysis. In addition, we gained six immune cells from the Tumor Immune Evaluation Resource (TIMER) website and analyzed the relationship between pyroptosis prognostic genes and immune infiltration.

**Result:**

Our results revealed that 31 DEPRGs were available by comparing normal and BLCA tissues with |log2 (fold change, FC)| > 0.5 and FDR <0.05. Four key genes (CRTAC1, GSDMB, AIM2, and FOXO3) derived from the pyroptosis prognostic model were experimentally validated for consistent expression in BLCA patients. Following risk scoring, the low-risk group of BLCA patients had noticeably higher overall survival (OS) than the high-risk group (*p* < 0.001). Risk score was still an independent prognostic factor (HR = 1.728, 95% CI =1.289–2.315, *p* < 0.001). In addition, we found remarkable correlations among the expression of pyroptosis-related prognostic genes and the immune infiltration of CD4^+^ T cells, CD8^+^ T cells, B cells, dendritic cells, macrophages, and neutrophils.

**Conclusion:**

Genes (CRTAC1, GSDMB, AIM2, and FOXO3) associated with pyroptosis are potential BLCA prognostic biomarkers that act as an essential part in the predictive prognosis of survival and immunotherapy of BLCA.

## Introduction

Bladder carcinoma (BLCA) is the prevalent malignancy of the urinary tract and the fourth most common malignancy in men, with a high mortality and morbidity rate (1, 2). In 2020, the number of new BLCA cases was predicted to be about 81,400, and the number of new deaths was calculated to be about 17,980 in the United States (3). Of these, non-muscle invasive bladder carcinoma (NMIBC), which is prone to recurrence, accounts for approximately 75%, and muscle invasive bladder carcinoma (MIBC) and metastatic disease, which are more aggressive, account for 25% (4, 5). Currently, cisplatin-based chemotherapy remains the first-line treatment option for advanced BLCA, but the response period to chemotherapy is limited and the prognosis of patients remains dismal, with a 5-year relative overall survival rate of only 15% for metastatic disease (5, 6). Moreover, studies have indicated that the wild-type TP53 gene expression profile was resistant to neoadjuvant chemotherapy in MIBC tumors (7). The highly heterogeneous and genomically unstable nature of BLCA have also been identified by an increasing number of studies (8, 9). The genetic prognostic biomarkers of BLCA have been emerging in recent years. An example was minichromosome maintenance protein 5 (MCM5), a biomarker of cell proliferation, which is expressed in normal urinary epithelium only in cells within the basal proliferative lumen and can be used to diagnose BLCA (10). A clinical trial demonstrated that the detection of MCM5 in urine sediment was a biomarker for predicting BLCA recurrence and prognosis. However, it was not widely used in clinical practice because its false-positive rate was higher than that of cystoscopy (11). Thus, searching for gene expression characteristics of BLCA patients and identifying newly effective prognostic markers are essential procedures for personalized treatment strategies to improve clinical outcomes.

Pyroptosis is a novel non-apoptotic mode of programmed cell death (PCD) mediated by the gasdermin family, characterized by pore formation on the cytoplasmic membrane, cell swelling, and rupture, and accompanied by the efflux of cell contents. Currently, the gasdermin family comprises six human homologs, gasdermin A–D (GSDMA–D), gasdermin E (GSDME, alternatively known as DFNA5), and Pejvakin (PJVK, alternatively known as DFNB59) (12). Pyroptosis can be induced by both the classical inflammasome pathway, which is activation of caspase-1 cutting the GSDMD protein, and the non-classical inflammasome pathway, which is bacterial lipopolysaccharide binding to caspase-4/5/11 cutting the GSDMD protein (13, 14). In addition, it was recently found that the serine protease granzyme A (GZMA) in cytotoxic lymphocytes could access target cells *via* perforin and evoke cancer cells to undergo pyroptosis by cleaving activated GSDMB protein, as well as activate immune response (15). Meanwhile, emerging lines of evidence also suggested that selective delivery of active gasdermin protein to cancer cells or targeted cleavage of GSDME by granzymes can inspire potent antitumor immunity (16, 17). Produced during pyroptosis, IL-1β and IL-18 can impair the recruitment of neutrophils, whereas GSDMD acted as an anti-inflammatory agent to stimulate neutrophil death (18). Moreover, GSDMD also increased cleavage in activated CD8^+^ T cells and enhanced their cytolytic ability (19). Indeed, immune infiltration of natural killer (NK), M1 macrophages, CD8^+^ T, and type 1 T helper (Th1) cells typically represented a good prognosis, while high-level infiltration of neutrophils, M2 macrophages, MDSCs, Treg cells, and Th2 cells was frequently correlated with a poor prognosis (20, 21). Interestingly, Hou J et al. demonstrated that GSDMC expression was correlated with breast cancer survival prognosis (22).

These studies have demonstrated that pyroptosis was inextricably bound to tumorigenesis, invasion, and tumor immune microenvironment, implicating that induction of tumor cell pyroptosis would be a potential strategy to strengthen the efficacy of anti-cancer therapy. Moreover, it has been determined that pyroptosis-related genes (PRGs) have been developed as markers of tumor prognosis in a variety of carcinomas, such as lung cancer (23), hepatocellular carcinoma (24), and breast cancer (25), by establishing prognostic models of pyroptosis genes. However, there was relatively less research on its specific functions in BLCA. Hence, we systematically researched and validated the expression levels of genes associated with pyroptosis in normal bladder and BLCA tissues to study the prognostic value of these genes. We also analyzed the relationship between PRGs and tumor immune microenvironment by the TIMER website.

## Materials and Methods

### Data Collection

**Figure 1:**
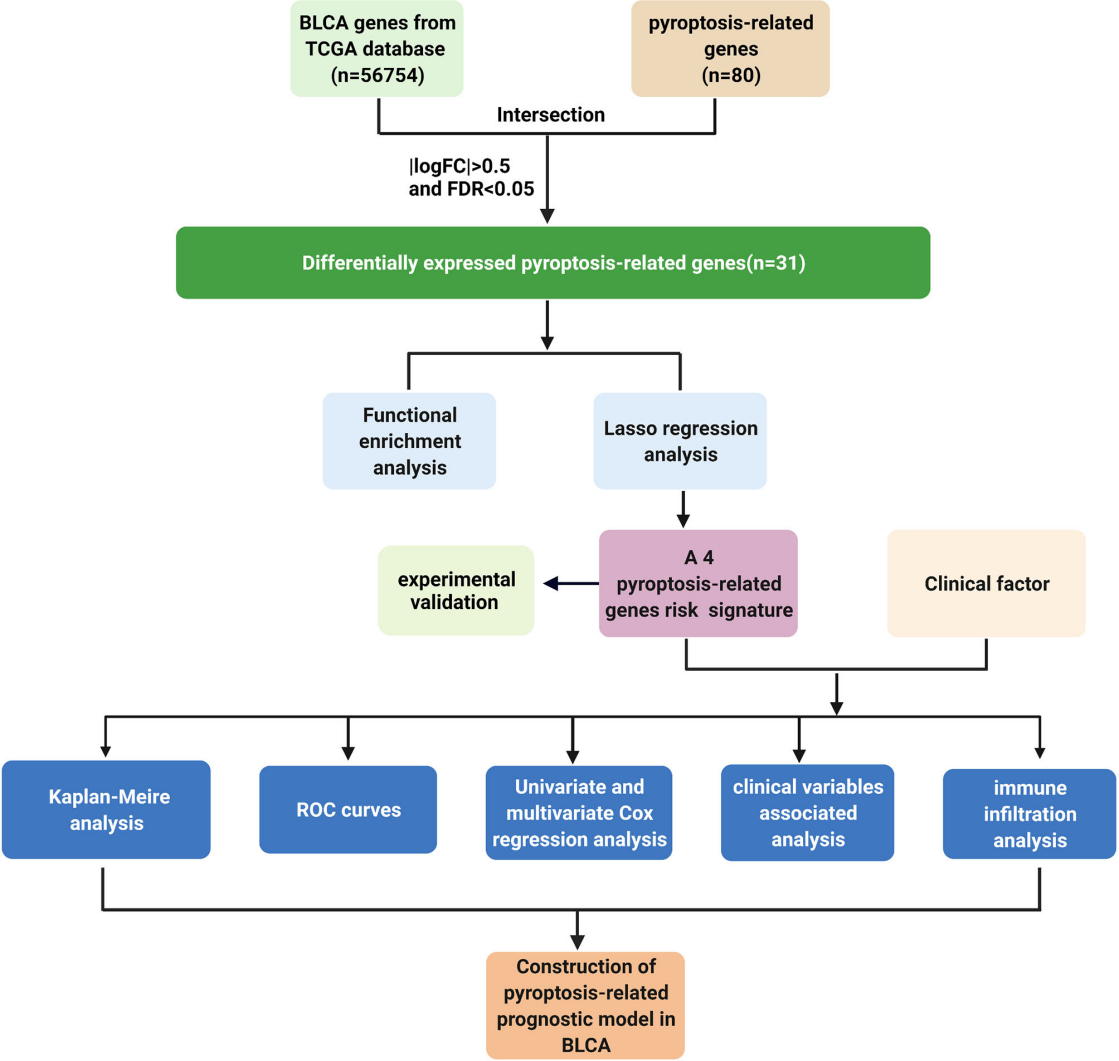
Workflow chart. Detailed data analysis workflow chart. Create BioRender.com.

As shown in the flowchart (**Figure 1**), search for the word “pyroptosis” AND “Homo sapiens” on the NCBI database (https://www.ncbi.nlm.nih.gov/gene/) to retrieve PRGs. About 80 pyroptosis genes were screened by this method. We obtained transcriptome data and relevant clinical profile of BLCA and normal samples from the TCGA database (https://portal.gdc.cancer.gov/).

### Enrichment Analysis of Differentially Expressed Pyroptosis-Related Genes

The condition |log2 (fold change, FC)| > 0.5 and FDR < 0.05 was established with R software to calculate the DEPRGs of BLCA vs. normal samples. Biological properties were analyzed by means of functional analysis, including Gene Ontology (GO) and the Kyoto Encyclopedia of Genes and Genomes (KEGG). We conducted biological process (BP), cellular component (CC), and molecular function (MF) enrichment analysis of DEPRGs using the R package “clusterprofler”. The same approach was also used for the enrichment analysis of KEGG. The *p*-value of less than 0.05 was statistically valid.

### Construction of Pyroptosis Prognosis Model Based on Pyroptosis-Related Genes

The DEPRGs expression signatures were intersected from the TCGA and NCBI databases. Univariate Cox regression analysis was applied to probe and identify the DEPRGs associated to survival of BLCA patients with a threshold value of *p* < 0.05 by the R package “survival”. Subsequently, the pyroptosis prognostic mode was structured and optimized to filter the key genes. Next, the risk score of individual patients was computed by the following equation: risk score = ∑X J * coef J, where coef J means coefficient, and X J means correlative expression of each DEPRGs normalized by Z-score. Finally, the median risk score categorized BLCA cases into two groups: high risk and low risk.

### Validation of the Key PRGs by Immunohistochemistry

To validate the pyroptosis prognosis model, we collected BLCA samples and adjacent non-cancerous bladder tissue from BLCA patients with primary surgical resection at Taizhou People’s Hospital. The Clinical Research Ethics Committee of Taizhou People’s Hospital has confirmed the tissues used in this study. Paraffin-embedded sections were stained for IHC analysis at 4 µm thickness. IHC was conducted with antibodies for CRTAC1 (1:200, bs-12948R, bioss), GSDMB (1:200, 12885-1-AP, Proteintech), AIM2 (1:200, PB9683, Boster), and FOXO3 (1:200, PB9196, Boster). Immunohistochemistry analysis was used following the manufacturer’s protocol. Then, two experienced pathologists, blinded to detailed information, would randomly select five regions and evaluate the immunostaining score.

### Prognostic Mode Evaluation and Clinical Relevance Assessment

Kaplan–Meier (K-M) survival and risk profile were depicted through the R package comparing the survival of the two groups. Next, we screened for separate prognostic factors by assessing the association between clinicopathological features and risk scores with the R package “Survival”. Finally, the ROC curve was calculated with the R package “survivalROC”, where the ROC area under the curve (AUC) value was analyzed to ascertain the prediction accuracy of the model. For clinical relevance, the scatter plot was pictured with the R package “beeswarm”. According to clinicopathological characteristics, key pyroptosis genes and risk value were analyzed to forecast the biological markers for the prognosis of BLCA.

### Analysis of Immune Cells Infiltration in PRGs of BLCA Patients

Systematic assessment of various immune cell infiltrations and their clinical influences can be conducted by TIMER (https://cistrome.shinyapps.io/timer/) (26). The relevance between PRG levels and immune cells infiltration was valued using the “Gene module”. For clinical outcomes, relevance to immune cells infiltration and PRG expression would be estimated by “Survival module”.

## Results

### Identification of DEPRGs Between Normal and BLCA Tissues

**Figure 2:**
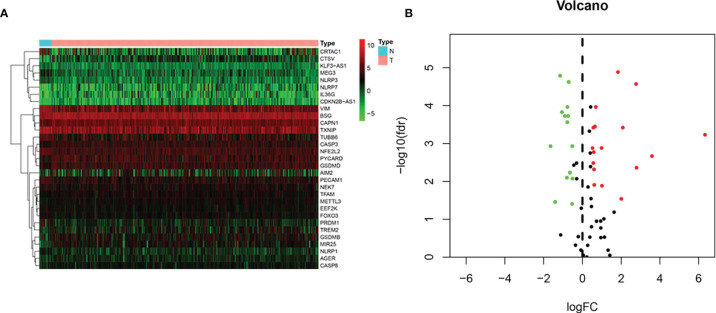
DEPRGs in BLCA versus non-cancerous samples. **(A)** Heatmap and **(B)** a volcano plot.

Gene expression profiles of 19 normal and 414 BLCA tissue samples and consequent clinical information were derived from the TCGA. Based on the search terms “pyroptosis” AND “Homo sapiens” in the Gene database, we identified 80 PRGs. Then, we found that 31 DEPRGs (Additional file 1) met the criteria of FDR < 0.05 and |log2(FC)| > 0.5 by R language screening, including 17 upregulated genes (CTSV, CAPN1, GSDMB, MIR25, PYCARD, TFAM, GSDMD, AIM2, NLRP7, IL36G, AGER, CDKN2B-AS1, METTL3, CASP3, TREM2, CASP8, and BSG) and 14 downregulated genes (VIM, PRDM1, TUBB6, MEG3, PECAM1, TXNIP, CRTAC1, KLF3-AS1, NEK7, NLRP3, EEF2K, FOXO3, NFE2L2, and NLRP1) (**Figures 2A, B**).

### Functional Enrichment Analysis of DEPRGs

**Figure 3:**
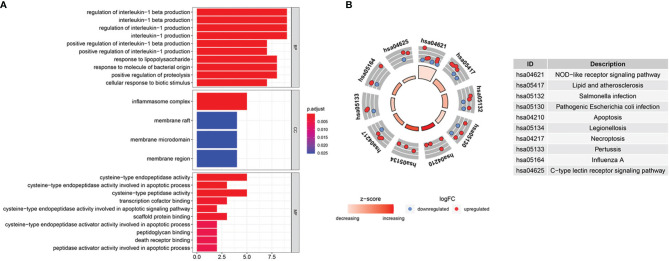
GO and KEGG enrichment analysis. **(A)** GO analysis of 31 DEPRGs. **(B)** The top 10 most enriched KEGG pathways analysis of 31 DEPRGs.

We scanned and analyzed the above 31 DEPRGs in BLCA with GO and KEGG to explore their potential signaling pathways. As shown in **Figure 3A**, BP indicated that DEPRGs were mainly enriched in the positive regulation of IL-1 and IL-1β production, response to LPS, etc. Among the CC, the most significantly altered pathway was the inflammasome complex. As for MF, cysteine-type endopeptidase activity, scaffold protein binding, and death receptor binding were significantly enhanced. The top 10 enriched signaling pathways with significant relevance in KEGG analysis were in the circle diagram (**Figure 3B**), including the NLR signaling pathway, apoptosis, necroptosis, and the C-type lectin receptor signaling pathway. Briefly, the analysis indicated a significant relevance between DEPRGs and the development of BLCA.

### Construction and Evaluation of Pyroptosis Prognosis Model in BLCA

**Figure 4:**
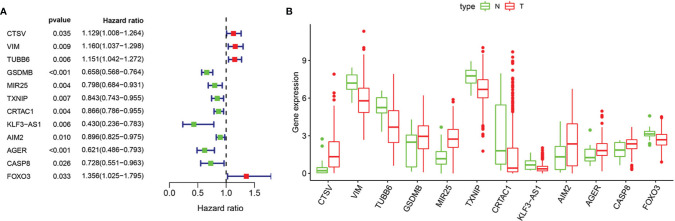
Regression analysis to select pyroptosis genes related to prognosis of BLCA. **(A)** Forest map of pyroptosis genes analyzed by univariate cox regression. **(B)** Boxplot of pyroptosis genes analyzed by LASSO regression. “N” stands for “Normal” and “T” stands for “Tumor”.

**Figure 5:**
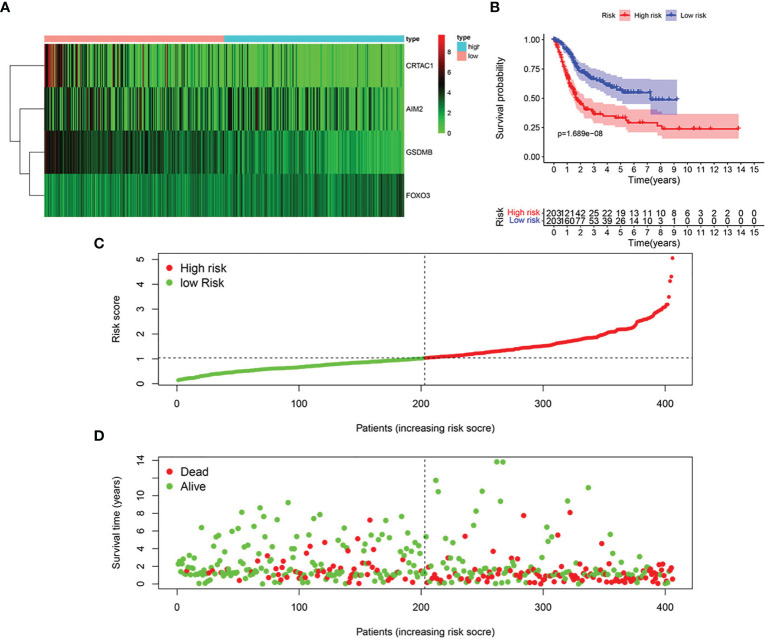
Structure of pyroptosis prognosis model in BLCA. **(A)** Heatmap displaying the expression patterns of those 4 key genes **(B)** K-M survival curve analysis. **(C)** Risk score distribution for BLCA cases. **(D)** OS for BLCA patients.

Based on univariate Cox regression analysis, we gained 12 genes associated with prognosis and structured a pyroptosis prognostic model (**Figure 4A**). The differential expression of 12 genes was displayed in the box plot of **Figure 4B**. Furthermore, the model was optimized by multivariate Cox regression analysis, and four key genes (CRTAC1, GSDMB, AIM2, and FOXO3) were obtained (**Table 1**). A heat map indicated the gene expression profiles of high- and low-risk groups in normal and BLCA (**Figure 5A**). Using K-M survival curve analysis, the 3- and 5-year survival rates for patients in the high- and low-risk group were 34.8% and 65.2%, and 31.1% and 54.8%, respectively. In a word, patients in group L had significantly higher survival rates relative to group H (*p* < 0.001) (**Figure 5B**). Risk score was computed for each patient based on the expression levels and risk factors of PRGs, and as shown in the risk curve results, the higher the risk score was, the shorter the survival time would be for BLCA patients (**Figures 5C, D**). It was suggested that these four DEPRGs may be potential prognostic indicators for BLCA.

**Table 1:** Genes included in prognostic gene signature.

Gene symbol	Full name	Coefficient	HR	*p*-value
GSDMB	Gasdermin B	−0.364568368	0.694496	1.11E-06
CRTAC1	Cartilage acidic protein 1	−0.127534411	0.880263	0.013359
AIM2	Absent in melanoma 2	−0.137029941	0.871944	0.002339
FOXO3	Forkhead box O3	0.281533186	1.32516	0.046972

### Expression Level of the Key PRGs in BLCA Tissues

**Figure 6:**
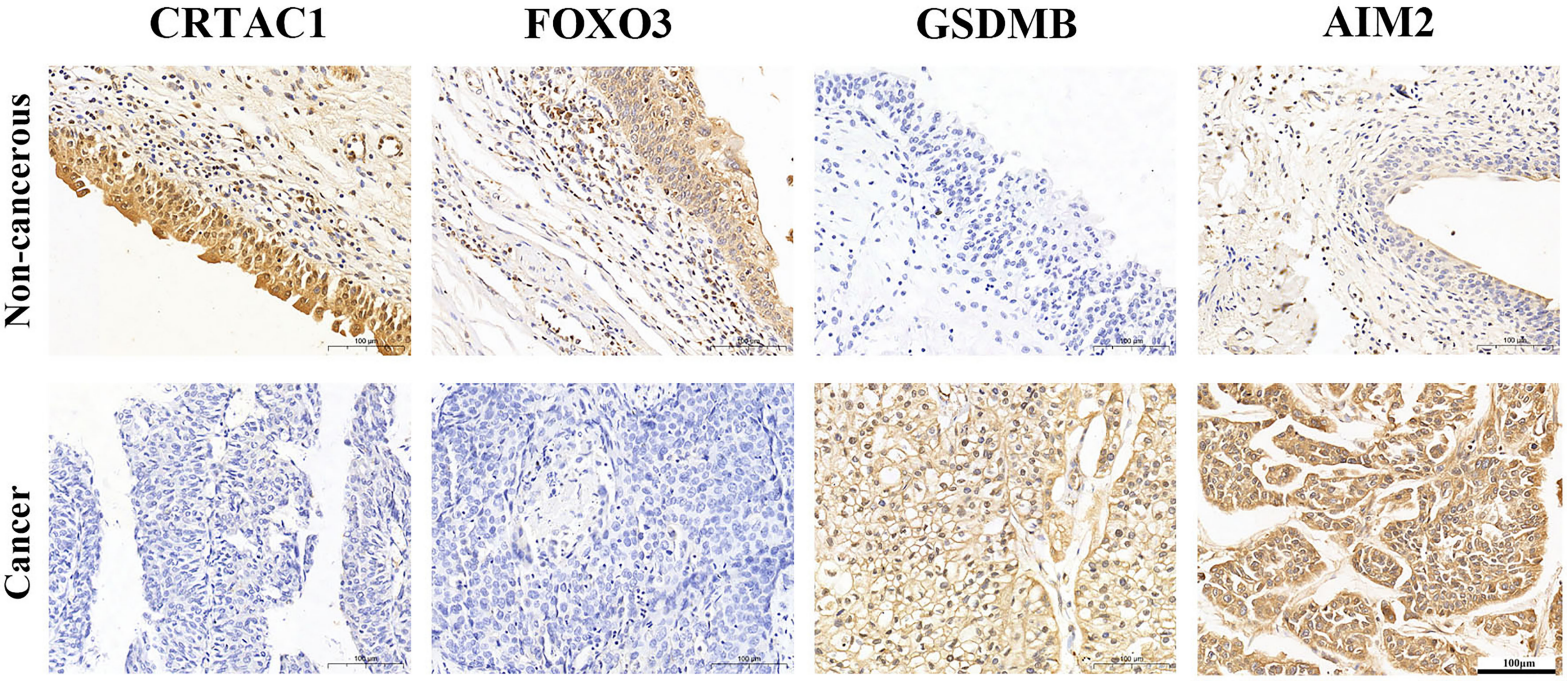
The expression protein of CRTAC1 FOXO3, GSDMB, and AIM2 in BLCA tissues and non-cancerous tissues (IHC).

IHC analysis confirmed that CRTAC1 and FOXO3 proteins were highly expressed in adjacent non-cancerous bladder tissues, 59.26% and 51.85%, respectively, while GSDMB and AIM2 proteins were highly expressed in BLCA tissues, 59.26% and 85.19%, respectively (**Figure 6**). Interestingly, it was consistent with the results of bioinformatics analysis, except that FOXO3 protein was also highly expressed in BLCA.

### Correlation Between Pyroptosis-Related Prognostic Genes and Clinicopathological Characteristics

**Figure 7:**
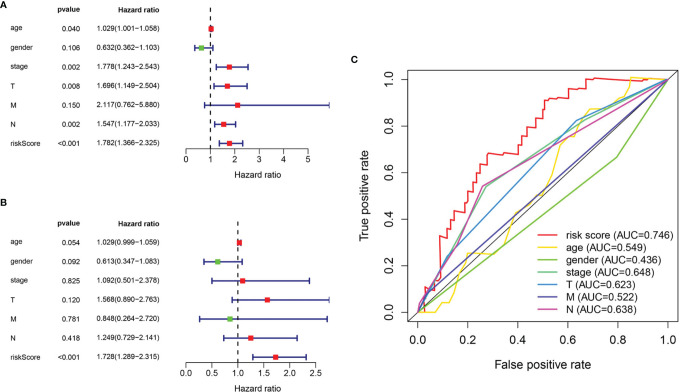
Evaluation of pyroptosis prognosis model in BLCA. **(A)** Univariate cox regression analysis. **(B)** Multiple cox independent prognosis analysis. **(C)** ROC curve of clinicopathological features.

The relationship between clinicopathological features and hazard scores was investigated by single and multifactorial Cox independent prognostic analyses. Univariate Cox regression analysis revealed that age, stage, T stage, N stage, and risk score were remarkably associated with BLCA OS (**Figure 7A**). The risk score was retained after multifactorial Cox regression analysis (**Figure 7B**). Furthermore, the accuracy of the model was assessed by combining risk scores with clinicopathological characteristics to picture ROC curves (**Figure 7C**). The larger the area under the ROC curve was, the more precise the prognostic model was structured.

**Figure 8:**
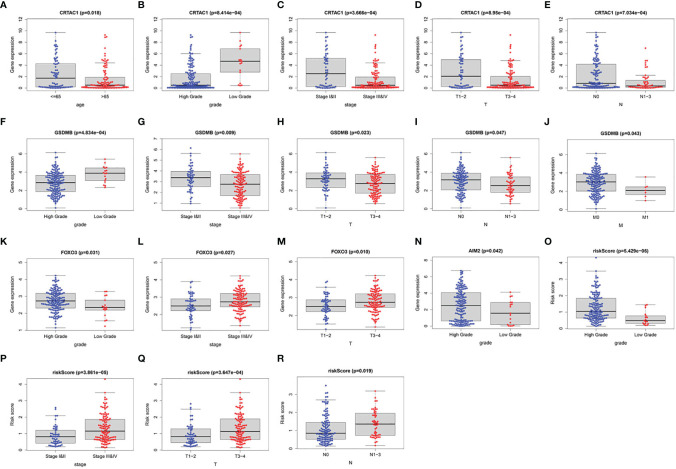
The clinical relevance analysis in BLCA. **(A–E)** CRTAC1 expression in ages, grade, pathological stages, pathological T and N stages. **(F–J)** GSDMB expression in grade, pathological stages, pathological T, N, and M stages. **(K–M)** FOXO3 expression in grade, pathological stages, pathological T stages. **(N)** AIM2 expression in grade. **(O–R)** Risk scores expressed in grades of pathological T and N stages.

Furthermore, we investigated the association of four prognostic genes (CRTAC1, GSDMB, AIM2, and FOXO3) and risk scores with clinical characteristics by independent samples *t*-test. As demonstrated in the scatter plot of **Figure 8**, decreased expression of CRTAC1 and GSDMB was significantly associated with advanced pathological T3–4, N1–3, and M1 stages, stages III and IV, and high grade (**Figures 8A–J**). Instead, high expression of FOXO3 and AIM2 was notably correlated with high grade, and overexpression of FOXO3 was also strongly correlated with stages III and IV and T3–4 (**Figures 8K–N**). Finally, we observed that higher risk values occurred in advanced pathological T3–4, late N1–3 stage, and higher stage (III and IV) (**Figures 8O–R**).

### Immune Cells Infiltration of Pyroptosis-Related Prognostic Genes in BLCA

**Figure 9:**
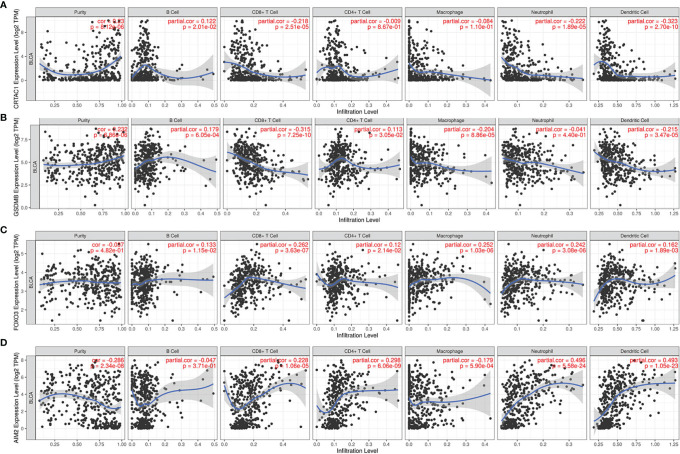
The relevance between pyroptosis-related prognostic genes and immune cell infiltration. The correlation between the abundance of immune cells and the expression of **(A)** CRTAC1, **(B)** GSDMB, **(C)** FOXO3, **(D)** AIM2 in BLCA.

It was demonstrated that pyroptosis participated in the modulation of the inflammatory response and tumor immune microenvironment. Therefore, we proceeded to comprehensively probe the association between DEPRGs in BLCA and immune cells infiltration. The results indicated that CRTAC1 expression was negatively relevant with the infiltration of CD8^+^ T cells (*p* = 2.51e−05), neutrophils (*p* = 1.89e−05), and dendritic cells (*p* = 2.70e−10) and positively relevant with the infiltration of B cells (*p* = 2.01e−02) (**Figure 9A**). Similarly, the expression of GSDMB was negatively associated with the infiltration of CD8^+^ T cells (*p* =7.25e−10), macrophages (*p* = 8.86e−05), and dendritic cells (*p* =3.47e−05) and positively associated with the infiltration of B cells (*p* = 6.05e−04) and CD4^+^ T cells (*p* = 3.05e−02) (**Figure 9B**). There was a positive relevance between FOXO3 expression and CD4^+^ T cells, CD8^+^ T cells, B cells, macrophages, dendritic cells, and neutrophils, all *p* < 0.05 (**Figure 9C**). In addition, there was a negative relevance between AIM2 expression and the infiltration of macrophages (*p* = 5.90e−04) and a positive association between AIM2 expression and the infiltration of CD8^+^ T cells (*p* = 1.06e−05), CD4^+^ T cells (*p* = 6.06e−09), neutrophils (*p* = 5.58e−24), and dendritic cells (*p* = 1.05e−23) (**Figure 9D**). Interestingly, FOXO3 and AIM2 showed opposite correlations with macrophages, which may be related to the lack of detailed typing of macrophages (M1 macrophages behaved as tumor suppressors, whereas M2 macrophages behaved as tumor promoters). In short, the infiltration of six types of immune cells (CD4^+^ T cells, CD8^+^ T cells, B cells, macrophages, dendritic cells, and neutrophils) was remarkably relevant with the expression of pyroptosis-related prognostic genes.

## Discussion

Pyroptosis is a lysis form of programmed death triggered by inflammasome and can rapidly release cellular contents such as pro-inflammatory molecules (IL-1β and IL-18) and antigens into the tumor microenvironment to activate the immune response of the body (27). A recent study has identified GSDM-mediated pyroptosis as a mechanism of cytotoxic lymphocyte killing to potentiate antitumor immunity (15). It was also confirmed that GSDME-mediated pyroptosis could promote T-cell recruitment through the release of relevant cytokines and trigger the activation of T cells to stimulate T-cell infiltration into tumor tissues (28). However, GSDME was often silenced in tumor tissues with promoter methylation, thus avoiding the occurrence of chemotherapeutic drug-induced pyroptosis and resisting antitumor effects (27, 29, 30). Hence, it is worthwhile to clarify the expression of PRGs in tumor tissues, and inducing pyroptosis in tumor cells would be possible and deemed as a robust method to promote the effect of oncotherapy.

With the development of bioinformatics and high-throughput sequencing technologies, it has become a reality to predict the prognosis of BLCA by assessing their risk level. For example, the risk models constructed based on m^6^A-related non-coding RNAs (31), pyroptosis-related non-coding RNAs (32), and ferroptosis-related genes (33), respectively, have evaluated the prognosis of BLCA patients or the response to immunotherapy. These models were constructed from different mechanisms to predict the prognosis of BLCA, yet unfortunately, the prognostic relationship and immune infiltration characteristics of pyroptosis-related mRNAs with BLCA patients were not considered.

In our study, we first explored the DEPRGs in normal and BLCA tissues, in which 31 genes were differentially expressed compared to normal tissue. These data suggested that differentially expressed genes may exert an underestimated role in the tumorigenesis and progression of BLCA. Then, we highlighted the function of DEPRGs using GO and KEGG pathway enrichment analysis. Predictably, we discovered that these genes were primarily relevant to the NLR signaling pathway, necroptosis, and regulation of IL-1β production. NLRs played an important regulatory role in inflammation-associated tumorigenesis. NLRs can specifically and sensitively recognize pathogen-associated molecular patterns, and activate caspase-1 or caspase-11, enhancing the production and release of IL-1β and IL-18 to recruit immune cells and promote the pyroptosis process (34, 35). Moreover, a study indicated that activation of the NF-κB/NLRP3/Caspase-1 pyroptosis signaling pathway exerted antitumor effects by increasing GSDMD levels and inhibiting cell proliferation and epithelial–mesenchymal transition progression (36). Therefore, DEPRGs may be potential targets for therapeutic and prognostic markers of BLCA.

Next, the expression of four key genes (CRTAC1, GSDMB, AIM2, and FOXO3) has been confirmed to be consistent with the bioinformatics analysis by IHC. However, the absence of difference in FOXO3 expression in cancerous and non-cancerous tissue may be related to both small sample size and the possibility that the FOXO3 gene was not transcribed in some adjacent non-cancerous bladder tissue. After that, they were screened to be associated with prognostic survival in BLCA by univariate and multifactorial Cox regression analysis. In addition, patient risk scores were calculated from mRNA expression levels and risk factors of each patient to obtain risk curves and K-M survival curves. Single- and multi-factor independent prognostic and clinical relevance analyses were conducted using the pyroptosis prognostic model to identify the relationship between gene expression levels and clinical prognosis. It was indicated that these genes were correlated with age, sex, grade, stage, and TNM subtype in the clinicopathological features (*p* < 0.05) and could be treated as a predictor of BLCA prognosis.

Cartilage acidic protein 1 (CRTAC1) is a glycosylated extracellular matrix molecule of human articular cartilage secreted by chondrocytes (37). They used gene expression data analysis from public databases to find that CRTAC1 expression was lower in lung adenocarcinoma (LUAD) than in normal tissues, demonstrating that the CRTAC1 gene was a protective gene in LUAD, and the high expression of CRTAC1 gene had a better prognosis (38). Equally, our study indicated that high expression of CRTAC1 was associated with earlier T (T1–2), N (N0) subtype, earlier stage (I and II), and low grades in BLCA patients. It suggested that the CRTAC1 gene could also be regarded as a protective gene in BLCA. Presently, there were no studies of CRTAC1 in BLCA or the relationship between CRTAC1 and pyroptosis, but our studies may provide new findings on prognostic markers in BLCA. The mechanism of CRTAC1 also needs further validation in BLCA.

GSDMB, as a member of the GSDM family, has also been observed to resemble the pyroptosis feature (39). However, the mechanism of GSDMB-induced pyroptosis was not quite clear and consistent. It was found that GSDMB-induced pyroptosis may be achieved by caspase-1 cleavage of GSDMB protein in 293T cells (40, 41). In contrast, Chen et al. demonstrated that GSDMB induced a conformational change in caspase-4 protein, which, in turn, increased caspase-4 enzyme activity and accelerated the cleavage of GSDMD, consequently causing non-canonical pyroptosis (42). In addition, the association between the high or low expression of GSDMB in normal and tumor tissues and prognosis was also controversial. Based on several studies (39, 43–45), hyperexpression of GSDMB in hepatocellular carcinoma, gastric cancer, and cervical cancer was considered as an oncogene that would appear to be involved in cancer invasion and metastasis. However, Shao Feng’s team indicated that CTLs could mediate target cell pyroptosis through GZMA cleavage of GSDMB in target cells. Respectively, they engrafted CT26 cells and B16-F10 cells with recombinant human GSDMB into mice subcutaneously, and found that GZMA/GSDMB pathway-mediated pyroptosis strengthened tumor clearance in the mouse model by enhancing the therapeutic efficacy of antitumor immunity. Meanwhile, they revealed that high GSDMB expression was relevant with OS benefit in BLCA patients (15). This is consistent with our findings. Our research also indicated that GSDMB was highly expressed in BLCA and hyper-expression of GSDMB was associated with low risk, low-grade infiltration, early stage (I and II), no lymph node infiltration (N0), and no distant metastasis (M0) of BLCA patients, all *p* < 0.05. Thus, GSDMB could serve as a protective prognostic biomarker for BLCA.

The forkhead box O class (FOXO) family is a widely expressed transcription factor, mainly including FOXO1, FOXO3, FOXO4, and FOXO6. The FOXO family is involved in several biological processes that were related to cell metabolism, cell proliferation, and apoptosis (46). Among them, FOXO3, also known as FOXO3a, plays a significant role in the regulation of cellular processes by targeting the expression and activity of effector genes. Meanwhile, the FOXO3 activity can be inhibited by an imbalance between kinases and phosphatases, significantly affecting cellular processes and even inducing carcinogenesis (47). The expression of FOXO3 in various tumors exhibited variable pro-carcinogenic and anti-carcinogenic effects. Qian et al. demonstrated that high expression of FOXO3 was relevant with the progression of glioblastoma, suggesting a poor prognosis for patients with glioblastoma (48), the same result for triple-negative breast cancer (49) and hepatocellular carcinoma (50). However, downregulation of FOXO3 promoted DNMT3B overexpression, leading to tumor growth in lung cancer, while clinical findings also showed that there was a high DNMT3B, low FOXO3a, and high MDM2 expression associated with low survival rates in lung cancer patients (51). Moreover, transcriptional inhibition of the FOXO3 gene directly and downregulation of FOXO3 protein phosphorylation promoted proliferation and migration of uroepithelial cancer cells *in vivo* and *in vitro* (52). Overexpression of circular RNA FOXO3 suppressed the proliferation, migration, and invasion of BLCA cell lines as well as promoted their apoptosis (53, 54). In contrast to our findings, FOXO3 was an indicator of poor prognosis in BLCA because it was enriched in high-risk groups, and the mechanism remains to be investigated. The study showed that downregulation of FOXO3 could inhibit NLRP3 inflammasome-mediated endothelial cell pyroptosis (55). However, the relationship between FOXO3 and pyroptosis in tumors has been rarely reported. Therefore, it is essential to further explore the regulation of FOXO3 on tumor cell pyroptosis.

Absent in melanoma 2 (AIM2), a typical receptor for cytoplasmic DNA contained a positively charged HIN-200 structural domain that recognized the sugar-phosphate backbone of host ectopic negatively charged double-stranded DNA (dsDNA) forming an electrostatic attraction (56). Host ectopic dsDNA activated AIM2 inflammasomes that drove the secretion of proinflammatory cytokines IL-18 and IL-1β, inducing cellular pyroptosis (57). AIM2 exhibited both growth inhibitory effects in colon, hepatoma, nasopharyngeal, and lung cancers, and proliferation-promoting effects in cutaneous squamous cell carcinomas (SCCs) and endometrial cancers (57–60). Compared with normal tissues, components of NLRP3 and AIM2 inflammasomes were highly expressed in nasopharyngeal carcinoma tissues, which correlated with an increased chance of survival in nasopharyngeal carcinoma (58). On the other hand, the absence of AIM2 inhibited the growth of cutaneous SCC (59). Therefore, the role of AIM2 in tumorigenesis was bipartite, and its outcome depended on the type of cancer. According to our findings, AIM2 appeared to be an oncogenic gene, as it was highly expressed in BLCA tissues and significantly upregulated in high-grade BLCA.

Briefly, all four genes (CRTAC1, GSDMB, FOXO3, and AIM2) were prognostic indicators of BLCA pyroptosis, with CRTAC1 and GSDMB suggesting a better prognosis, and FOXO3 and AIM2 suggesting a poor prognosis.

PRGs worked through cell membrane pore formation to activate pyroptosis, enhance antitumor immunity, and inhibit tumor growth. Expression of pyroptosis genes strengthened phagocytosis of tumor cells by tumor-associated macrophages (TAM), as well as the count and function of tumor-infiltrating NK cells (17). However, TAM infiltration in BLCA disrupted the adaptive immune response and promoted tumor vascularization, which, in turn, led to tumor progression (61). Meanwhile, one study described that BLCA can be stratified as high or low risk on the basis of immune cells status (62). It was evident that the infiltration of immune cells was of great importance to the survival prognosis of BLCA. It was shown that BLCA recruited more T cells than normal bladder cells, and that infiltrating T cells increased the proliferation and invasion of BLCA cells by modulating the ERβ/c-MET or ERβ/IL-1/c-MET signaling pathways, promoting metastasis (63). Neutrophils may promote tumor aggressiveness and progression in BLCA owing to their stimulation of excessive inflammation, release of growth factors to malignant cells, and secretion of human neutrophil polypeptides (64). Within the present research, we indicated a remarkable relevance between the expression of PRGs (CRTAC1, GSDMB, FOXO3, and AIM2) and the infiltration of six types of immune cells, including CD4^+^ T cells, CD8^+^ T cells, B cells, macrophages, dendritic cells, and neutrophils, implying that these genes not only could serve as prognostic indicators, but also may reflect the immune status.

Chen et al. (65) have previously constructed a pyroptosis-related prognostic model to predict survival in BLCA patients, although their study contained only 53 pyroptosis genes compared with 80 pyroptosis genes in our study. Moreover, there was a lack of experimental validation in their study. Unfortunately, our study also has some limitations. It is unclear how these DEPRGs in the prognostic model we constructed modulate the underlying mechanisms of the BLCA process. A further exploration of their biological functions is required by elaborate experiments. Furthermore, this was a retrospective study, and we would like to conduct further well-designed prospective studies to confirm our findings.

## Conclusion

In summary, our study has constructed a prognostic model based on pyroptosis in BLCA and identified four key prognostic genes (CRTAC1, GSDMB, FOXO3, and AIM2), which also reflected immune status. We expect that our findings will provide new insights into emerging immunotherapeutic targets, identify biomarkers to more precisely predict survival in BLCA patients, and provide data to support clinical formulation of personalized treatment plans.

## Data Availability Statement

The datasets presented in this study can be found in online repositories. The names of the repository/repositories and accession number(s) can be found in the article/**Supplementary Material**.

## Ethics Statement

The studies involving human participants were reviewed and approved by the Clinical Research Ethics Committee of Taizhou People’s Hospital. Written informed consent for participation was not required for this study in accordance with the national legislation and the institutional requirements.

## Author Contributions

Conception: CY, QG, and XY. Equally contributing to the paper, study design, data analysis, experiment execution, and drafting: JY and HL. Experiment execution: YW. Study supervision: PF and YZ. Final approval: all authors. All authors contributed to the article and approved the submitted version.

## Funding

This research was supported by the Taizhou Science and Technology Support Plan (Social Development) project (TS201903), the China Postdoctoral Science Foundation (2019M663505), and the Postdoctoral Interdisciplinary Innovation Foundation, Sichuan University (No. 0040204153243).

## Conflict of Interest

The authors declare that the research was conducted in the absence of any commercial or financial relationships that could be construed as a potential conflict of interest.

## Publisher’s Note

All claims expressed in this article are solely those of the authors and do not necessarily represent those of their affiliated organizations, or those of the publisher, the editors and the reviewers. Any product that may be evaluated in this article, or claim that may be made by its manufacturer, is not guaranteed or endorsed by the publisher.
